# Effectiveness of baloxavir marboxil in nonhuman primates infected with highly pathogenic avian influenza A(H7N9) virus

**DOI:** 10.1016/j.ebiom.2026.106350

**Published:** 2026-06-25

**Authors:** Kiyoko Iwatsuki-Horimoto, Masaki Imai, Toru Ishibashi, Shinya Yamada, Maki Kiso, Mutsumi Ito, Atsuhiro Yasuhara, Seiya Yamayoshi, Yoshihiro Kawaoka

**Affiliations:** a Division of Virology, Institute of Medical Science, University of Tokyo, Tokyo, Japan; b The University of Tokyo Pandemic Preparedness, Infection and Advanced Research Center (UTOPIA), University of Tokyo, Tokyo, Japan; c International Virus Infectious Disease Research Center, National Institute of Global Health and Medicine, Japan Institute for Health Security, Tokyo, Japan; d Department of Special Pathogens, International Research Center for Infectious Diseases, Institute of Medical Science, University of Tokyo, Tokyo, Japan; e Clinical Pharmacology & Pharmacokinetics, Medical Science Department, Shionogi & Co., Ltd., Osaka, Japan; f Department of Pathobiological Sciences, School of Veterinary Medicine, University of Wisconsin-Madison, Madison, WI, USA

**Keywords:** Influenza virus, HPAI, A(H7N9), Nonhuman primate, Baloxavir marboxil

## Abstract

**Background:**

Highly pathogenic avian influenza (HPAI) A(H7N9) virus poses a potential public health threat, underscoring the need for effective antiviral options for outbreak preparedness. Baloxavir marboxil (BXM) is a cap-dependent endonuclease inhibitor approved for seasonal influenza, but its in vivo efficacy against HPAI A(H7N9) virus has not been fully evaluated.

**Methods:**

We evaluated the efficacy of BXM in cynomolgus macaques infected with a reverse genetics-generated HPAI A(H7N9) virus. Animals received either low- or high-dose BXM, single-dose oseltamivir, or vehicle at 4 or 48 h post-infection (hpi). BXM administration was designed to mimic human pharmacokinetics. Viral titres, body temperature, body weight, lung pathology, and treatment-emergent viral substitutions were analysed.

**Findings:**

Early treatment (4 hpi) with BXM significantly reduced viral titres in nasal and tracheal swabs, lessened weight loss, and decreased pulmonary inflammation and alveolar damage compared to untreated or oseltamivir-treated animals. Virus pathogenicity was relatively mild; no animals died. Delayed treatment (48 hpi) showed limited benefit. The PA-I38T (83.8%) and PA-E23G (78.6%) substitutions associated with BXM resistance were detected in one animal, and a PA-K34R (85.4%) substitution was detected in another animal. These substitutions reduce BXM susceptibility and were detected at low titres.

**Interpretation:**

Although the dosing regimen used in this study involved repeat dosing to achieve the plasma drug concentrations after a single dose in humans, these findings highlight the importance of early antiviral intervention and support BXM use as a potential countermeasure against HPAI A(H7N9) virus infection, as resistance-associated substitutions remained limited in the macaque model. BXM may be a valuable therapeutic option for HPAI A(H7N9) virus infections.

**Funding:**

Supported by the Japan Agency for Medical Research and Development (JP20wm0125002, JP223fa627001) and Shionogi & Co., Ltd.

**Research in context**

**Evidence before this study**

We searched PubMed for studies published until May 2026 that included terms such as “baloxavir”, “baloxavir marboxil (BXM)”, “oseltamivir”, “H7N9”, “highly pathogenic avian influenza”, “macaque”, “non-human primate”, and “antiviral”. We included relevant studies published in English or Japanese that investigated the antiviral efficacy of antiviral agents against influenza viruses in vitro, in animal models, or in clinical settings.

BXM is a cap-dependent endonuclease inhibitor approved for the treatment of seasonal influenza; it has shown potent antiviral activity against a range of influenza A viruses in vitro and in animal models. However, evidence for its in vivo efficacy against highly pathogenic avian influenza (HPAI) A(H7N9) viruses, particularly in non-human primate models that closely reflect human disease, has been limited. In addition, concerns remain regarding the emergence and biological significance of BXM resistance-associated substitutions during treatment.

**Added value of this study**

This study provides in vivo evidence in a cynomolgus macaque model that early administration of BXM significantly reduces viral replication, body weight loss, and lung pathology following HPAI A(H7N9) virus infection. When tested in macaques infected with the same virus challenge dose, BXM showed greater antiviral efficacy than oseltamivir under the experimental conditions used. Although treatment-emergent PA substitutions associated with reduced BXM susceptibility were detected, they occurred at low frequencies and did not compromise overall antiviral efficacy.

**Implications of all the available evidence**

Our findings support the use of BXM as a potential therapeutic option for HPAI A(H7N9) virus infections. They also clarify the benefits of early antiviral intervention and the limited impact of resistance-associated substitutions in vivo, supporting consideration of BXM stockpiling for future avian influenza outbreaks.

## Introduction

Low pathogenic avian influenza (LPAI) A(H7N9) viruses have caused human infections since they were first identified in China in 2013.^1^, ^2^, ^3^, ^4^ While these viruses cause mild or asymptomatic disease in poultry, they can cause severe infections in humans, with a fatality rate of approximately 41% as of February 2017.^5^ In 2017, the LPAI A(H7N9) viruses acquired substitutions responsible for high pathogenicity in the multi-basic cleavage site of their haemagglutinin (HA) protein, which increased morbidity and mortality in infected poultry in China.^6^, ^7^, ^8^, ^9^ Although the overall severity of highly pathogenic avian influenza (HPAI) A(H7N9) infection of patients was similar to that of LPAI A(H7N9) infections,^3^^,^^10^^,^^11^ the HPAI A(H7N9) viruses caused a greater number of confirmed human cases.^12^ In addition, in Japan, HPAI A(H7N9) viruses were isolated from duck meat products carried illegally by passengers from China and relinquished voluntarily at the border,^13^^,^^14^ highlighting the potential risk of international introduction of these viruses. Since its emergence, avian influenza A(H7N9) virus has caused more than 1500 laboratory-confirmed human infections with a high case-fatality rate, and although no human cases have been reported since 2019, as of March 2026,^15^ these viruses continue to be reported in poultry to the World Organisation for Animal Health. It has been reported that 23% of human cases of HPAI A(H7N9) infection treated with oseltamivir developed neuraminidase (NA) inhibitor resistance mutations.^16^ To address public health concerns, more treatment options for HPAI A(H7N9) virus infections are needed.

Baloxavir marboxil (BXM) is an orally available prodrug that is rapidly converted after administration to its active form, baloxavir acid (BXA), which targets the viral endonuclease activity of the influenza polymerase acidic (PA) protein.^17^^,^^18^ BXM was approved in Japan and other countries, including the United States, in 2018. The clinical efficacy of BXM was demonstrated by its ability to rapidly reduce viral loads compared to the NA inhibitor oseltamivir phosphate.^19^^,^^20^ Oseltamivir is one of the most widely prescribed antivirals and remains a standard of care for influenza treatment; however, NA protein substitutions that reduce susceptibility to oseltamivir have been reported, including in viruses of the A(H7N9) subtype.^21^, ^22^, ^23^ Reduced susceptibility to BXM has also been reported, due to amino acid substitutions in the PA protein, including PA-I38X and PA-E23X, as well as other substitutions such as PA-K34R, PA-A37T, and PA-E199G.^19^^,^^20^^,^^24^, ^25^, ^26^ Previously, Suzuki et al. assessed the efficacy of BXM against HPAI A(H7N9) virus in cynomolgus macaques^27^ and found that virus titres in macaques treasted with BXM were significantly lower than those in animals treated with NA inhibitors (i.e., oseltamivir or zanamivir). The average histological pneumonia scores at 8 days post-infection (dpi) in the treated groups tended to be lower than those in the control group, although the difference was not statistically significant.^27^ Suzuki et al. administered BXM to the macaques as single dose of 1 mg/kg on day 1 after infection. However, pharmacokinetic studies have shown that plasma concentrations of BXA decline more rapidly in macaques than in humans.^28^ Therefore, in the present study, we administered BXM to cynomolgus macaques at plasma concentrations of BXA consistent with those in humans and analysed the effects of the drug and the emergence of BXM-resistant viruses.

## Methods

### Ethics

All experiments in cynomolgus macaques were approved by the Institutional Animal Care and Use Committee of Shin Nippon Biomedical Laboratories, Ltd. (Approval No. IACUC814-010), and were performed in accordance with the animal welfare bylaws of Shin Nippon Biomedical Laboratories, Ltd., Drug Safety Research Laboratories, which is fully accredited by AAALAC International. This study was also conducted in accordance with the ARRIVE guidelines.

### Cells and viruses

Madin–Darby canine kidney (MDCK) cells were maintained in Eagle's MEM containing 5% newborn calf serum and incubated at 37 °C with 5% CO_2_. The MDCK cells used in this study were from a laboratory stock,^29^ and their identity was confirmed by DNA fingerprinting analysis, which demonstrated profiles indistinguishable from those of the standard ATCC cell line controls (Supplementary Table S1). HPAI influenza A/Guangdong/17SF003/2016-NA294R virus (A(H7N9); GD/3) which possesses a multi-basic motif at its HA cleavage site, was created by reverse genetics; it possess arginine at position 294 in NA (N9 numbering), consistent with the predominant residue identified in the parental virus stock, which contained a mixed population at this position, including a minor population associated with resistance to NA inhibitors. This virus was generated in the background of the consensus sequence of the remaining GD/3 genome and was amplified in MDCK cells (GISAID accession no. EPI_ISL_20450171).^17^ Plasmid-based reverse genetics for influenza virus generation was performed as previously described.^30^ Their genomic sequences were confirmed by Sanger sequencing. All experiments with A(H7N9) viruses were performed in enhanced biosafety level 3 (BSL3) containment laboratories at the University of Tokyo and Shin Nippon Biomedical Laboratories, Ltd., which are approved for such use by the Ministry of Agriculture, Forestry and Fisheries, Japan.

### Preparation of antiviral compounds

BXM (S-033188, provided by Shionogi & Co., Ltd., Osaka, Japan) is an orally available prodrug that is rapidly converted to its active form, BXA (S-033447).^31^^,^^32^ Oseltamivir phosphate (provided by Shionogi & Co., Ltd., Osaka, Japan), which is rapidly converted to its active metabolite oseltamivir carboxylate, was used as a comparator drug.^33^ A 0.5 w/v% methylcellulose (MC) solution (Fujifilm Wako Pure Chemical Industries, Ltd.) was used as the vehicle control.

BXM and oseltamivir were weighed without prior pulverisation and transferred to a conditioning mixer. The appropriate amount of vehicle (approximately 4 ml per g of test compound) was added, and the mixture was processed using a conditioning mixer with vacuum capabilities to obtain a homogeneous suspension. The suspension was then transferred to a measuring cylinder and diluted with vehicle to prepare the final formulations. BXM doses were based on previously reported single-dose pharmacokinetics data in cynomolgus macaques^28^ to achieve plasma concentrations comparable to those in humans, while the oseltamivir dose was higher than previously reported efficacious doses in macaques.^34^

### Animals

We used approximately 2–3-year-old male cynomolgus macaques (*Macaca fascicularis*) that were negative in virus neutralisation assays for A (H1N1)pdm09, A (H1N1), A (H3N2), B-Victoria lineage, and B-Yamagata lineage viruses infection, and serologically negative for influenza virus infection based on an Anti-Influenza virus A IgG Human ELISA kit (abcam plc, Cambridge, UK) using anti-Monkey IgG HRP (Funakoshi Co., Ltd, Tokyo, Japan) as the secondary antibody (Supplementary Table S1). Only male cynomolgus macaques were included in this study to reduce variability within the limited sample size and to maintain consistency across experimental groups. Animals were housed under standardised conditions at Shin Nippon Biomedical Laboratories, Ltd. (Kagoshima, Japan) throughout the 7- or 8-day acclimatisation and experimental periods, with a temperature range of 23–29 °C, relative humidity of 30%–70%, and a 12-h light/dark cycle. Environmental enrichment was provided, including continuous access to toys, food-based enrichment (pieces of apple or sweet potato supplied at least five times weekly), and daily cleaning and disinfection of the enclosure floor. No specific additional measures were taken to control for potential confounders such as treatment or measurement order, or animal/cage location beyond standard husbandry and experimental procedures. No formal blinding was implemented the during outcome assessment or data analysis. No expected or unexpected adverse events occurred during the study.

### Experimental infection and antiviral compound treatment of cynomolgus macaques

Animal experiments were conducted by Shin Nippon Biomedical Laboratories, Ltd. (Kagoshima, Japan). Anaesthesia was induced with concomitant intramuscular administration of ketamine hydrochloride (0.1 ml/kg, 5 mg/kg; Arevipharma GmbH, 50 mg/ml or Ketalar for Intramuscular Injection 500 mg, Daiichi Sankyo Propharma Co., Ltd., 50 mg/ml) and medetomidine hydrochloride (0.08 ml/kg, 0.08 mg/kg; Domitor, Orion Corporation, 1 mg/ml). Under anaesthesia, cynomolgus macaques were inoculated with GD/3 (10^7^ plaque forming unit (PFU)/ml) through a combination of the intratracheal (4.5 ml), intranasal (0.5 ml per nostril), ocular (0.1 ml per eye) and oral (1 ml) routes, resulting in a total infectious dose of 6.7 × 10^7^ PFU as previously described.^35^, ^36^, ^37^, ^38^ At 4- or 48-h post-infection (hpi), macaques were administered a low- or high-dose of BXM, single-dose oseltamivir phosphate as a comparator, or MC solution as a control vehicle (details of dosing are provided in Table 1, and Supplementary Table S2). The formulations were administered directly into the stomach via a disposable catheter inserted through the nasal cavity with a syringe in conscious animals under gentle restraint, once daily for 5 days. Clinical signs (see Supplementary Table S3 for details) were examined twice daily from day 0 to day 9 [once in the morning (before dosing on dosing days) and once in the afternoon (after dosing on dosing days)] for all animals, and once daily from day 10 onward. Plasma was collected from blood drawn from the femoral vein with a syringe containing heparin sodium at 24, 48, 72, 96, and 120 h after the first dose for pharmacokinetic assessments in conscious animals under gentle restraint. To assess virus growth, nasal and tracheal swabs were collected on days 1, 3, 5, 7 (n = 6) and on day 9 (n = 3) before dosing on dosing days. On day 7, three animals were euthanised and nasal turbinates and lungs were collected. Swabs were suspended in PBS containing 0.1% BSA and penicillin-streptomycin, and organs were homogenised with MEM containing 0.3% BSA. Both swab suspensions and organ homogenates were titrated in MDCK cells by using plaque assays. The remaining animals (n = 3) were observed daily for clinical signs, and their body weight and rectal temperature were measured once on days 0, 1, 3, 5, 7, 9, 12, 15, 18, and 21. These animals were euthanised at 21 dpi and sera were collected for antibody titre measurements. Excised macaque lung tissues were fixed in 10% neutral buffered formalin, and processed for paraffin embedding for pathological study. Humane endpoints, including criteria such as body weight loss and clinical signs, were predefined in accordance with institutional guidelines and approved by the Institutional Animal Care and Use Committee of Shin Nippon Biomedical Laboratories; however, none of these endpoints were reached during the study.

**Table 1:** Concentration of antiviral compounds.

Designation	Compound	Dose (mg/kg)					Volume (mL/kg)
		1st	2nd	3rd	4th	5th	
Low-dose BXM^a^	Baloxavir marboxil	4.5	1.5	1.2	0.8	0.6	5
High-dose BXM^b^	Baloxavir marboxil	7.0	2.8	2.1	8.6	4.0	5
Oseltamivir	Oseltamivir phosphate	100	100	100	100	100	5
Vehicle	0.5% Methylcellulose solution	–	–	–	–	–	5

a Low-dose BXM, which mimics plasma concentration after a single human dose.

b High-dose BXM, which mimics plasma concentration after two human doses.

### Pharmacokinetics

The pharmacokinetic simulation was performed using Phoenix WinNonlin (Certara, Randor, PA).^39^ To determine the concentration baloxavir acid (BXA; S-033447), the active metabolite of BXM (S-033188), plasma was added to 0.1% formic acid in acetonitrile for virus inactivation and deproteinisation. To the resultant supernatants, 1 mg/ml EDTA solution and internal standard solution were added and mixed and the samples were then subjected to liquid chromatography with tandem mass spectrometry (LC/MS/MS) analysis. LC/MS/MS analysis was performed using an API5000 system (Sciex, Framingham, MA). The method was validated over a range of 0.5–500 ng/ml, with a lower limit of quantification (LLOQ) of 0.5 ng/ml.

### Plaque assay

Confluent monolayers of MDCK cells were washed with MEM containing 0.3% BSA, infected with diluted homogenates, and incubated for 30–60 min at 37 °C. After the inoculum was removed, the cells were washed with MEM containing 0.3% BSA and overlaid with a 1:1 mixture of 2x MEM/0.6% BSA and 2% agarose containing 1 μg/ml tosylsulfonyl phenylalanyl chloromethyl ketone (TPCK)-trypsin. Plates were incubated at 37 °C for 48 h before virus plaques were counted. Area under the curve (AUC) values were calculated from virus titres over time using GraphPad Prism 9.

### Pathological examination

Pathological examinations and evaluations were conducted by Sept. Sapie CO., Ltd. (Tokyo, Japan). Paraffin blocks were sectioned at a thickness of 4 μm using standard histological techniques. The sections were stained using a routine haematoxylin and eosin (HE) procedure. For immunohistochemical (IHC) analysis, serial sections were deparaffinised and subjected to antigen retrieval using Proteinase K (DAKO Agilent Technology, Tokyo, Japan) for 10 min at room temperature. Endogenous peroxidase activity was blocked with 3% hydrogen peroxide for 5 min. The sections were then incubated with a rabbit monoclonal antibody against type A influenza virus nucleoprotein (Clone 010, Sino Biological, Kanagawa, Japan, Supplementary Table S1) at 1:250 for 50 min at room temperature, followed by incubation with Histofine Simple stain MAX-PO MULTI (Nichirei Bioscience, Tokyo, Japan). Immunoreactivity was visualised using a diaminobenzidine chromogen, and counterstaining was performed with haematoxylin. Histopathological evaluation was conducted in a blinded manner by a board-certified pathologist (Hisashi Yoshimura, DVM, Ph.D., Diplomate JCVP).

### Deep sequence

Viral RNA was extracted by using a QIAamp Viral RNA Mini Kit (QIAGEN) or the Maxwell® System (Promega). The PA and NA segments of influenza A virus were simultaneously amplified by using the SuperScript IV one-step RT-PCR system (Thermo Fisher Scientific) with specific primers [MBTuniPA-13 (5′-ACG CGT GAT CAG TAG AAA CAA GGT ACT T-3′) and MBTuniPA-12-R (5′-ACG CGT GAT CAG CRA AAG CAG GTA C-3′) and MBTuniN9-13 (5′-ACG CGT GAT CAG TAG AAA CAA GGG TCT T-3′) and MBTuniN9-12-R (5′-ACG CGT GAT CAG CAA AAG CAG GGT C-3′)]. The PCR products were processed for sequencing libraries by using a QIAseq FX DNA Library Kit (QIAGEN) and were then analysed by using the MiSeq i100 Plus System (Illumina) with the MiSeq i100 Series 25M Reagent Kit (300 cycles). To determine the virus sequences, the reads were assembled by CLC Genomics Workbench (version 25, Qiagen) with GD/3 as a reference.

### Antiviral susceptibility testing

To determine polymerase inhibitor susceptibility, MDCK cells in 6-well plates were infected with the indicated virus that resulted in approximately 50 plaques per well. After an incubation for 60 min at 37 °C, the viral inoculum was removed and the cells were overlaid with MEM containing 1% agarose, 0.3% bovine serum albumin, 1 μg/ml tosylsulfonyl phenylalanyl chloromethyl ketone (TPCK)-treated trypsin, and different concentrations of BXM. The cells were then incubated for 3 days at 37 °C. Then, the cells were fixed with 10% formalin, overlays were removed, plates were dried, and plaques were counted. The 50% effective concentration (EC_50_) values were calculated by using Graphpad Prism.

To determine NA inhibitor susceptibility, virus dilutions (equivalent to 800 to 1200 fluorescence units) were mixed with oseltamivir carboxylate (0.01 nM–1 mM) in 2-(N-morpholino) ethanesulfonic acid containing calcium chloride and incubated for 30 min at 37 °C. Then, methylumbelliferyl-N-acetylneuraminic acid was added as a fluorescent substrate and incubated for 1 h at 37 °C. Sodium hydroxide in 80% ethanol was added to the mixture to stop the reaction. The fluorescence of the solution was measured at an excitation wavelength of 360 nm and an emission wavelength of 465 nm. EC_50_ values were calculated by using Graphpad Prism.

### Statistics

Sample sizes were based on our previous studies with the macaque model. On the final day of the 7–8-day acclimation period, animals were assigned to groups by stratified randomisation using the MiTOX System (Mitsui Zosen Systems Research Inc.) according to body weight, to minimise differences in body weight among groups. No animals or data points were excluded from the experiments or analyses, and no a priori inclusion or exclusion criteria were established. GraphPad Prism 9 software was used for all statistical analyses. No statistical method was used to predetermine sample size. Normality and homogeneity of variance were assessed prior to statistical analyses, and when assumptions were not met, appropriate alternative tests were applied. A *P* value less than 0.05 was considered statistically significant. All data are presented as means ± s.e.m. Statistical comparisons between groups and over time were performed using the tests indicated in the figure legends (mixed-effects analysis with Dunnett's multiple comparisons test vs. control for Fig. 2, 1-way ANOVA with Tukey's multiple comparisons test for Fig. 3, and Brown–Forsythe test and Welch's ANOVA with Dunnett's T3 multiple comparisons test for Fig. 4). Differences were considered statistically significant as indicated in the figure legends.

### Role of funders

The Japan Agency for Medical Research and Development (provided funding through JP20wm0125002 and JP223fa627001) had no role in the study design, data collection, analysis, interpretation, or manuscript preparation. Shionogi & Co., Ltd. supplied BXM (S-033188) and oseltamivir phosphate and performed plasma concentration analysis.

## Results

### Clinical parameters in macaques infected with HPAI A(H7N9) virus and antiviral treatment

**Fig. 1:**
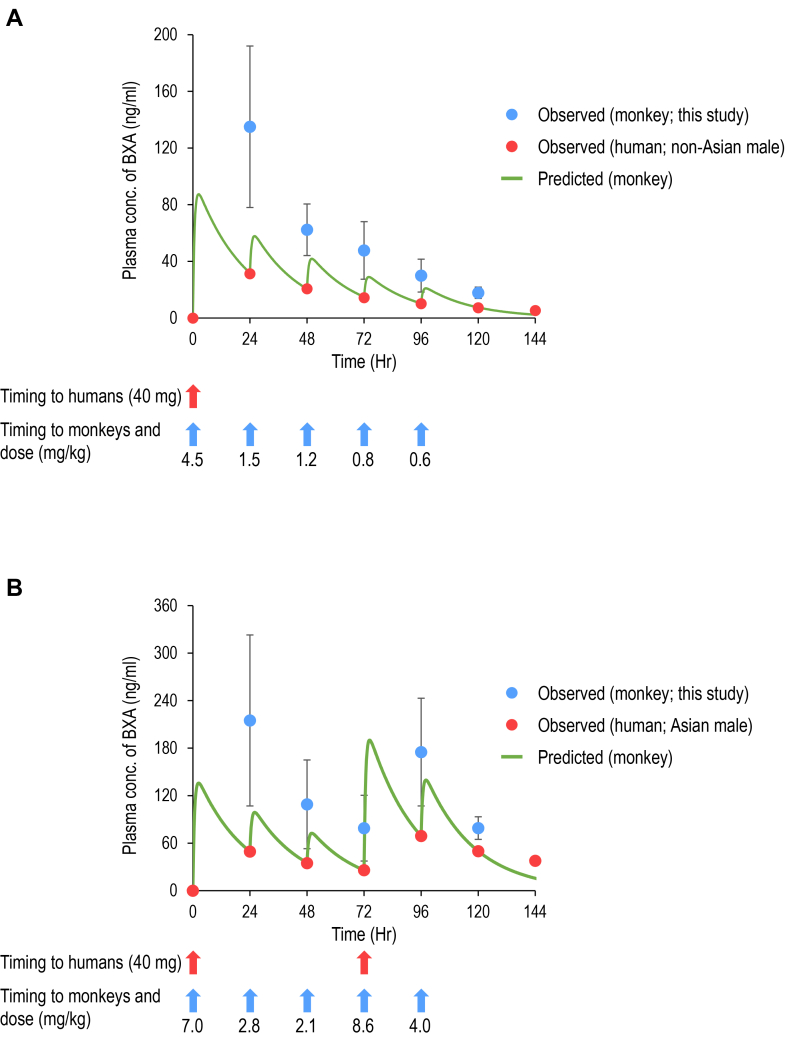
**Plasma concentrations of BXA in macaques after oral dose of BXM.** The pharmacokinetics of BXA following oral administration of low (A; n = 3, animals #40–42) or high (B; n = 3, animals #43–45) doses of BXM in macaques, corresponding to those in a human administered one dose (non-Asian male; 67.7 kg) or two doses (Asian male; 67.7 kg), were simulated based on previous BXA pharmacokinetic data in macaques.^28^ Red dots indicate the pharmacokinetics of BXA in a human. Green lines indicate the simulated pharmacokinetics in a macaque. Blue dots indicate the actual pharmacokinetics in macaques in this study (Data are means ± s.e.m.). The timings and doses of drug administration for a human (red arrows) and for monkeys (blue arrows) are shown at the bottom of the graph.

**Fig. 2:**
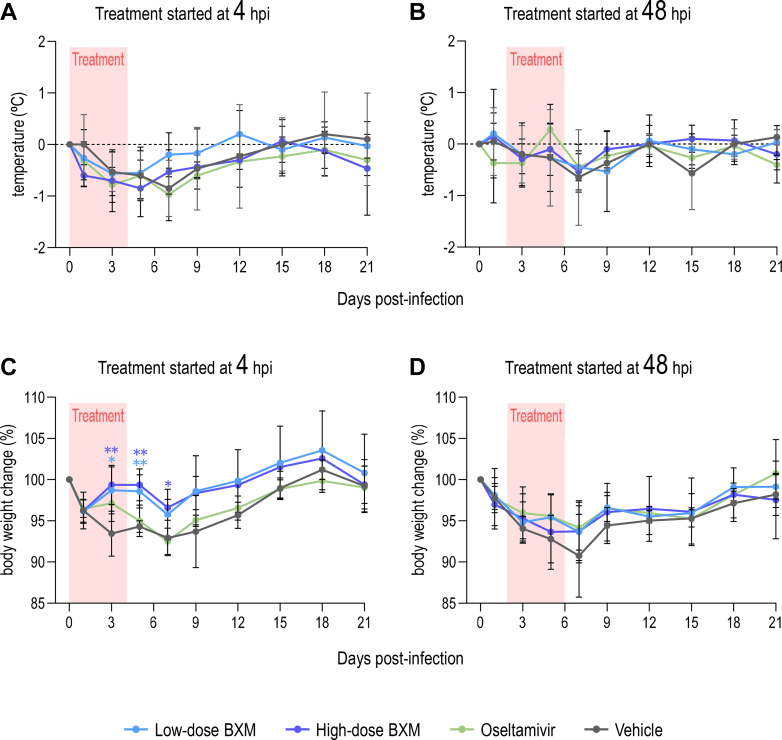
**Clinical changes in HPAI A(H7N9) virus-infected and treated macaques.** Cynomolgus macaques (n = 6 to 7 dpi, n = 3 from 9 dpi) were inoculated with GD/3 and treatment was initiated at 4 hpi (A, C) or 48 hpi (B, D). The pink background indicates the duration of treatment. (A, B) Average body temperatures were compared with that on Day 0 before virus inoculation, which were set as 0 °C to represent changes in temperature over time. (C, D) The body weight changes of macaques were monitored. Body weights of individual animals are shown as the percentage of the body weight compared with that on Day 0. Data are means ± s.e.m. (mixed-effects analysis with Dunnett's multiple comparisons test vs. control; ∗ *P* < 0.05; ∗∗ *P* < 0.01). Light blue: low-dose BXM group, dark blue: high-dose BXM group, green: oseltamivir group, grey: control group.

Approximately 2–3-year-old male cynomolgus macaques (n = 6 per group) were anaesthetised and inoculated with 6.7 × 10^7^ PFU/animal of HPAI GD/3 A(H7N9) virus via multiple routes. At 4- or 48-hpi, treatment of the macaques was initiated once a day for 5 days using a low-dose or a high-dose of BXM, oseltamivir phosphate, or MC solution as the control vehicle (see Table 1). The BXM doses were determined by simulating the plasma BXA concentration profiles based on previous pharmacokinetic data from a single dose in macaques^28^ to mimic a plasma BXA concentration profile after a single dose in a non-Asian male (67.7 kg) (Fig. 1A), or two doses in an Asian male (67.7 kg) (Fig. 1B). For pharmacokinetic assessment of BXA, the active metabolite of BXM, blood was drawn at 24, 48, 72, 96, and 120 h after the first dose. Although the observed plasma concentrations of BXA were higher than those reported in humans, the pharmacokinetic trends in macaques and humans were similar (Fig. 1) when BXM was given as scheduled. As a control, single-dose oseltamivir phosphate was administered at 100 mg/kg/day, which exceeds the dose previously reported to achieve pharmacologically effective plasma concentrations in cynomolgus macaques (30 mg/kg/day),^34^ taking into consideration the high replicative ability of the virus. Clinical signs and body weights were monitored for 7- (n = 3) or 21- (n = 3) dpi (Supplementary Table S2). Only one macaque, #37, in the untreated vehicle group showed clinical signs, a decrease in spontaneous activity, and delayed awakening from anaesthesia, at 5 dpi (Supplementary Table S5). The other macaques showed no clinical signs (Supplementary Tables S4 and S5). No significant changes in body temperature were observed in any of the macaque groups, including the untreated group (Fig. 2A and B). Average body weight loss was observed in all groups except the groups that initiated treatment with high- or low-dose BXM at 4 hpi; there were significant differences in body weight loss on days 3 and 5 post-infection for the low-dose group and on days 3, 5, and 7 post-infection for the high-dose group compared to the untreated group (Fig. 2C). In contrast, when treatment was initiated at 48 hpi, body weight loss was observed in all groups, with no significant differences among them (Fig. 2D). Thus, when treatment was started at 4 hpi, BXM was effective in preventing the reduction in weight loss at both the low and high dose.

### Virus titres in swab samples

**Fig. 3:**
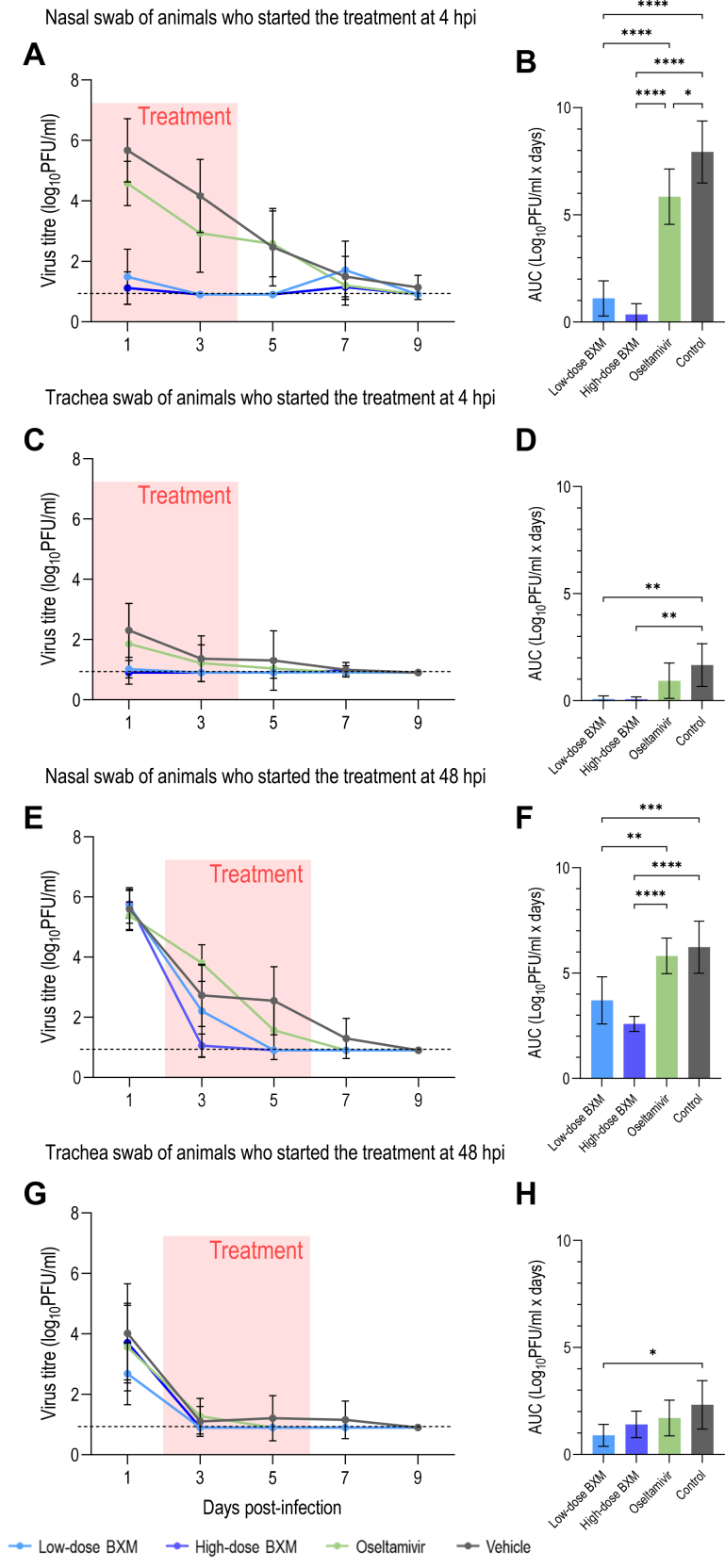
**Virus titres in swabs from HPAI A(H7N9) virus-infected and treated macaques.** Cynomolgus macaques (n = 3) were inoculated with GD/3 and treated. (A–D) Nasal and tracheal swabs were collected from macaques whose treatment started at 4 hpi. (E–H) Nasal and tracheal swabs were collected from macaques whose treatment started at 48 hpi. Left panels show averages of virus titres in the nasal (A and E), and tracheal (C and G) swabs for the individual titres listed in Table 1. Right panels show AUC values of the virus titres in the nasal (B and F), and tracheal (D and H) swabs calculated on the basis of the individual titres listed in Table 1. Data are means ± s.e.m. (1-way ANOVA with Tukey's multiple comparisons test; ∗ *P* < 0.05; ∗∗ *P* < 0.01, ∗∗∗ *P* < 0.001, ∗∗∗∗ *P* < 0.0001). The pink background indicates the duration of treatment. Light blue: low-dose BXM group, dark blue: high-dose BXM group, green: oseltamivir group, grey: control group.

Virus titres in nasal and tracheal swabs of macaques infected with GD/3 were examined. When treatment was initiated at 4 hpi, virus was detected in nasal swabs in the control and oseltamivir groups for up to 9 and 7 dpi, respectively (Fig. 3A, Table 2). Similarly, virus was detected in tracheal swabs from both the control and oseltamivir groups for up to 7 and 5 dpi, respectively (Fig. 3C, Table 2). In contrast, in the BXM groups, virus was detected in only a few macaques: at 1 dpi, 2 of 6 in the low-dose group and 1 of 6 in the high-dose group were virus-positive, and no virus was detected at 3 and 5 dpi. However, at 7 dpi, virus was detected in 3 of 6 macaques in the low-dose group and in 1 of 6 in the high-dose group (Fig. 3A and C, Table 2). Some of the viruses detected at 7 dpi showed reduced BXA susceptibility with PA substitutions (see below). When treatment was initiated at 48 hpi, virus was detected in nasal and tracheal swabs of macaques in both the control and oseltamivir groups for up to 5 dpi and 3 dpi, respectively. In both the high- and low-dose BXM groups, virus was detected in the nasal and tracheal swabs for up to 3 and 1 dpi, respectively (Fig. 3E and G, Table 3).

**Table 2:** Virus titres in swabs from H7N9 virus-infected cynomolgus macaques that began treatment 4 hpi.

swab	Day	Virus titres (log_10_ PFU/ml) in swab samples collected from animals infected and treated with:																							
		Low-dose BXM						High-dose BXM						Oseltamivir						Vehicle					
		#4	#5	#6	#16	#17	#18	#7	#8	#9	#19	#20	#21	#10	#11	#12	#22	#23	#24	#1	#2	#3	#13	#14	#15
Nasal swab	1	<^a^	<	<	2.68	2.66	<	<	<	<	2.20	<	<	4.53	5.77	5.11	3.92	3.89	4.26	4.07	5.32	5.53	7.32	5.87	5.90
	3	<	<	<	<	<	<	<	<	<	<	<	<	4.81	3.15	3.68	1.00	2.30	2.63	3.32	4.45	4.10	2.78	4.04	6.30
	5	<	<	<	<	<	<	<	<	<	<	<	<	2.81	<	3.43	1.60	3.08	3.63	<	3.86	3.18	<	2.6	3.38
	7	<	2.51	3.13	<	1.90	<	<	<	<	<	2.38^e^	<	1.70	<	1.90	<	<	<	<	<	1.78	1.00	2.51	1.90
	9	<	<	<	–b	–	–	<	<	<	–	–	–	<	<	<	–	–	–	<	<	1.60	–	–	–
Tracheal swab	1	<	<	<	1.60	<	<	<	<	<	<	<	<	3.43	<	3.72	<	<	1.30	1.60	2.63	2.74	1.00	2.32	3.53
	3	<	u.d.^c^	<	<	<	<	<	<	<	<	<	<	<	<	2.41	<	<	1.30	<	2.71	1.85	<	<	<
	5	<	<	<	<	<	<	<	<	<	<	<	<	<	<	<	<	<	1.70	<	<	<	<	<	3.32
	7	<	<	<	<	1.00^d^	<	<	<	<	<	1.30^f^	<	<	<	<	<	<	<	<	<	<	<	<	1.48
	9	<	<	<	–	–	–	<	<	<	–	–	–	<	<	<	–	–	–	<	u.d.	<	–	–	–

a <, virus not detected (detection limit; <1 log_10_ PFU/ml).

b –, no samples were collected.

c u.d., undecidable due to bacterial growth.

d PA-K34R (85.4%).

e PA-E23G (78.6%).

f PA-I38T (83.8%).

**Table 3:** Virus titres in swabs from A(H7N9) virus-infected cynomolgus macaques that began treatment 48 hpi.

swab	Day	Virus titres (log_10_ PFU/ml) in swab samples collected from animals infected and treated with:																							
		Low-dose BXM						High-dose BXM						Oseltamivir						Vehicle					
		#28	#29	#30	#40	#41	#42	#31	#32	#33	#43	#44	#45	#34	#35	#36	#46	#47	#48	#25	#26	#27	#37	#38	#39
Nasal swab	1	6.41	5.04	5.52	6.23	5.79	5.15	5.71	6.08	6.36	5.85	5.04	5.51	5.09	5.43	6.06	5.64	4.70	5.36	5.32	6.89	5.64	4.70	5.32	5.72
	3	3.15	<	4.83	1.78	<	1.70	<	<	1.85	<	<	<	3.34	3.04	4.57	4.30	3.45	4.11	3.32	2.34	4.36	1.30	2.72	2.34
	5	<^a^	<	<	<	<	<	<	<	<	<	<	<	3.05	1.00^d^	2.59	<	1.00	<	3.26	3.36	<	3.28	3.20	1.30
	7	<	<	<	<	<	<	<	<	<	<	<	<	<	<	<	<	<	<	<	<	<	2.49	1.70	<
	9	<	<	<	–b	–	–	<	<	<	–	–	–	<	<	<	–	–	–	<	<	<	–	–	–
Tracheal swab	1	2.48	2.59	4.60	1.70	1.95	2.78^c^	4.83	3.38	3.36	3.41	1.94	5.38	3.97	4.74	5.64	2.53	2.51	2.00	4.65	6.90	3.92	2.56	3.62	2.43
	3	<	<	<	<	<	<	<	<	<	<	<	<	1.00	2.34	<	<	1.60	<	<	2.11	<	<	<	<
	5	<	<	<	<	<	<	<	<	<	<	<	<	<	<	<	<	<	<	<	<	<	2.74	<	<
	7	<	<	<	<	<	<	<	<	<	<	<	<	<	<	<	<	<	<	<	<	<	2.43	<	<
	9	<	<	<	–	–	–	<	<	<	–	–	–	<	<	<	–	–	–	<	<	<	–	–	–

a <, virus not detected (detection limit; <1 log_10_ PFU/ml).

b –, no samples were collected.

c PA-E319G (55.7%); PA-K539R (30.6%).

d NA-I26T (46.4%); NA-G133R (31.0%).

To quantitatively compare the viruses recovered from the swabs, the area under the curve (AUC) of the virus titre was calculated (Fig. 3B, D, F, and H). When treatment was started at 4 hpi, the AUC of the nasal swabs was significantly lower for both the low- and high-dose BXM groups compared to the oseltamivir and control groups. Additionally, the oseltamivir group showed a modest but significant reduction compared to the control group (Fig. 3B). Similarly, the AUC of the tracheal swabs was significantly lower in both BXM groups compared to the control group (Fig. 3D). When treatment was started at 48 hpi, the AUC of the nasal swabs was significantly lower both for the low- and high-dose BXM group compared to the oseltamivir and control groups, but there was no significant difference between the oseltamivir and control groups (Fig. 3F). In the AUC of the tracheal swabs, the low-dose BXM group showed a modest but significant reduction compared to the control group, whereas no significant difference was observed for the high-dose BXM group (Fig. 3H). Thus, virus titres in macaques treated with BXM were significantly lower than those in the other groups.

### Detected amino acid substitutions and drug susceptibility

All swab samples in which virus was detected were analysed by deep sequencing. For the group in which treatment with BXM was initiated at 4 hpi, PA-K34R (85.4%) was present in the tracheal swab of animal #17 at 7 dpi; in addition, PA-E23G (78.6%) was present in the nasal swab and PA-I38T (83.8%) in the tracheal swab of animal #20 at 7 dpi (Table 2). The I38T, E23G, and K34R substitutions in PA are known to reduce susceptibility to BXM.^26^

For the group in which treatment with low-dose BXM was initiated at 48 hpi, virus from the tracheal swab of animal #42 at 1 dpi contained a mixture of PA-E319G (55.7%) and PA-K539R (30.6%). These substitutions have not been linked to BXM resistance, and this virus disappeared by 3 dpi (Table 3). In the oseltamivir group, virus from the nasal swab of animal #35 at 5 dpi contained a mixture of NA-I26T (46.4%) and NA-G133R (31.0%). All of these viruses retained the multi-basic motif at the HA cleavage site and maintained their high pathogenicity. These substitutions have not been linked to oseltamivir resistance, and this virus disappeared by 7 dpi (Table 3).

To determine drug susceptibility, we generate these mutant viruses by reverse genetics. For substitutions observed as a mixture in vivo, we constructed viruses carrying each single substitution as well as a virus carrying both substitutions. We found that viruses carrying PA-I38T were much less susceptible to BXM, as previously reported, and that PA-E23G and PA-K34R also caused reduced susceptibility consistent with prior studies (Table 4). In contrast, PA-E319G, PA-K539R, and the PA-E319G plus PA-K539R combination of substitutions did not confer resistance to BXM. Regarding oseltamivir, NA-I26T did not reduce susceptibility, whereas NA-G133R alone and the NA-I26T plus NA-G133R combination could not be analysed due to insufficient viral replication (Table 4). Taken together, these results confirm that PA-I38T confers high-level resistance and that PA-E23G and PA-K34R also affect susceptibility.

**Table 4:** Susceptibility to BXM and oseltamivir of A(H7N9) viruses carrying treatment-emergent PA and NA substitutions.

Substitution(s)	BXM (fold-difference)^b^	Oseltamivir (fold-difference)
PA-E23G	3.3 (5.59)^a^	–c
PA-K34R	9.9 (16.78)^a^	–
PA-I38T	360 (610.17)^a^	–
PA-E319G	0.72 (1.22)	–
PA-K539R	0.49 (0.83)	–
PA-E319G + K539R	1.6 (2.71)	–
NA-I26T	–	5.6 (1.10)
NA-G133R	–	u.d.^d^
NA-I26T + G133R	–	u.d.
WT	0.59	5.1

EC_50_ values (nM) to antivirals were measured in duplicate by using a plaque reduction assay for BXM or a sialidase inhibition assay for oseltamivir.

a Substitutions associated with a >3-fold reduction in susceptibility to the antiviral were considered resistance-associated substitutions.

b Fold-difference compared with WT, inoculated GD/3 HPAI A(H7N9) virus.

c –, not tested.

d u.d., undetermined due to insufficient viral replication.

### Virus replication and pathogenicity in organs

**Fig. 4:**
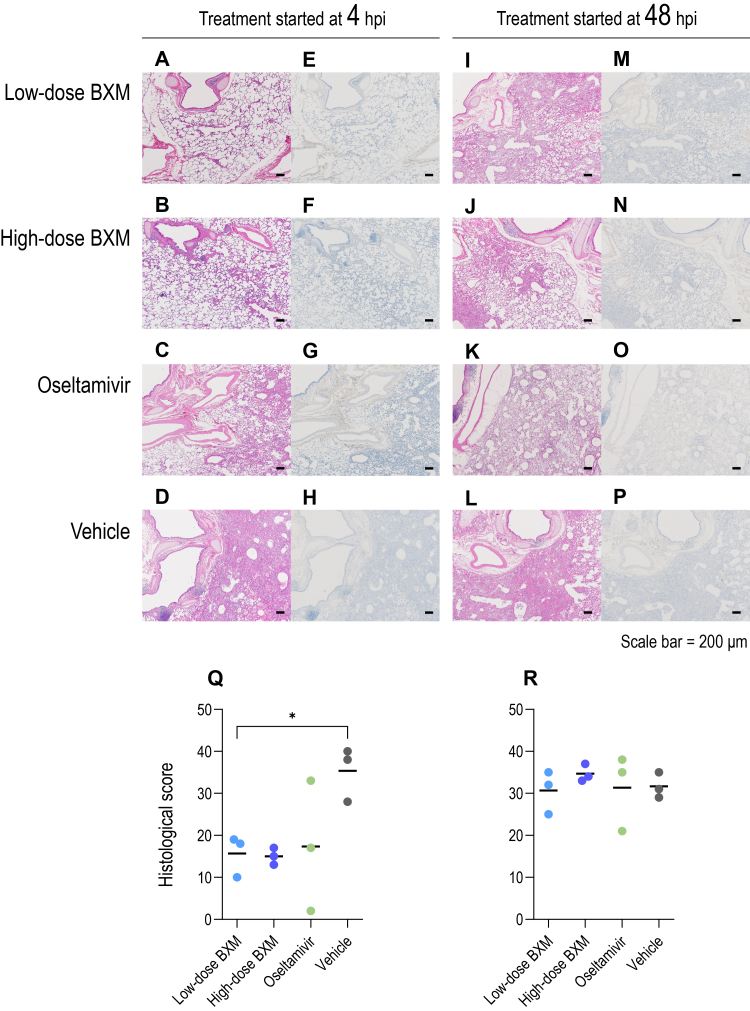
**Pathological examination of the lungs of macaques.** The images show the haematoxylin and eosin staining (left panel) and immunohistochemical analysis using an anti-influenza A virus nucleoprotein antibody (right panel) analysis of the lungs of macaques at 7 dpi in the low- (A, E, I, M) or high-dose (B, F, J, N) BXM, oseltamivir (C, G, K, O), or control (vehicle) (D, H, L, P) groups, with treatment started at 4 hpi (A–D, E–H) or 48 hpi (I–L, M−P). Representative images are shown; panels A and E correspond to animal #18, B and F to #19, C and G to #22, D and H to #13, I and M to #41, J and N to #43, K and O to #46, and L and P to #37. Scale bars = 200 μm. Histopathological pneumonia scores for groups treated starting at 4 hpi (Q) and 48 hpi (R) based on the degree of inflammatory cell infiltration in the bronchi, peribronchiolar areas, alveolar spaces, and perivascular regions. The low-dose BXM group for which treatment started at 4 hpi showed significantly reduced inflammation compared to the control (vehicle) group (Brown–Forsythe test and Welch's ANOVA with Dunnett's T3 multiple comparisons test; ∗ *p* < 0.05).

At 7 dpi (n = 3), macaques were euthanised and their organs were collected for virological and pathological examination. Although virus was detected in nasal swabs from some of the animals in the oseltamivir and control groups for up to 7dpi and 9 dpi, respectively (Table 2), virus was recovered from the respiratory organs of only two macaques in the control group (trachea and bronchus of #37, trachea of #39; 5.21, 4.64, and 5.70 log_10_ PFU/g, respectively). Except for animal #24, positive immunostaining was sporadically detected in at least one of the following cell types: bronchial epithelial cells, bronchiolar epithelial cells, alveolar epithelial cells, and alveolar macrophages. However, the number of positive cells was limited in all animals, and there were no marked differences between the groups (Fig. 4E–H, M–P; Supplementary Table S6). On pathological examination, when treatment was started at 4 hpi, animals in the low- and high-dose BXM groups (Fig. 4A and B), and in the oseltamivir group (Fig. 4C) exhibited less inflammatory cell infiltration around the bronchi and bronchioles compared to the control group (Fig. 4D). One animal in the oseltamivir-treated group, #24, showed no lung lesions or viral antigen detection (Supplementary Fig. S1), although infection was established in nasal and tracheal tissues (See Table 2). In addition, animals in the low- and high-dose BXM groups showed fewer alveolar lesions (Fig. 4A and B) compared to the control group (Fig. 4D). In contrast, when treatment was started at 48 hpi, no histological differences in the degree of lesions were observed between any of the groups (Fig. 4I–L). The degree of pulmonary inflammatory response was scored based on the extent of inflammatory cell infiltration in the bronchi, peribronchiolar areas, alveolar spaces, and perivascular regions, as well as the density of inflammatory cells present. The mean histopathological pneumonia scores showed no statistically significant difference between the control and treatment groups when treatment was initiated at 48 hpi (Fig. 4R). However, when treatment was started at 4 hpi, animals in the low-dose BXM-treated group exhibited significantly lower inflammation scores compared to the vehicle control group (Fig. 4Q). Taken together, these results demonstrate the therapeutic value of early administration of BXM in reducing lung inflammation and viral replication.

## Discussion

In this study, we administered BXM to influenza virus-infected macaques such that the drug concentration mimicked that used in humans, because drug clearance in macaques is faster than in humans. Similar differences have been reported in mice^40^^,^^41^ and ferrets,^42^ in which BXA is also cleared more rapidly than in humans, supporting the need to adjust dosing regimens in non-human primate models to approximate clinically relevant plasma exposures. We found that virus titres in macaques treated with BXM were significantly lower than those in the other experimental groups. Furthermore, pathological examination revealed that in the groups where treatment started at 4 hpi, both the low- and high-dose BXM groups exhibited less inflammatory cell infiltration around the bronchi and bronchioles, as well as significantly fewer alveolar lesions compared to the control group. These results indicate that BXM is highly effective against HPAI A(H7N9) virus when used early after infection. In contrast, delayed treatment (i.e., initiated at 48 hpi) limited the antiviral efficacy. These results indicate that the timing of treatment initiation plays a more important role than dosage in determining the efficacy of BXM. Early administration of antiviral drugs is known to be vital for seasonal influenza treatment with oseltamivir,^43^^,^^44^ and this also applies to the use of BXM against A(H7N9) virus. The maximum benefit of BXM is exerted when the drug is administered early during infection, underscoring the importance of prompt diagnosis and antiviral intervention.

The pathogenicity of GD/3 A(H7N9) virus in macaques was not particularly severe in this study, and no fatal cases were observed, similar to our previous study.^17^ In contrast, Suzuki et al. reported mortality among A(H7N9) HPAI virus-infected macaques, although the strain they used was different and the titres of infected virus were lower than ours.^27^ Although the exact reason for the discrepancy between the two studies remains unclear, it is possible that differences in the viral strain may have influenced the outcome. Compared to the study by Suzuki et al.,^27^ both the pathogenicity and the dosage and timing of BXM administration differed from ours (1 mg/2 ml/kg and once on day 1 after infection). In their study, Suzuki et al.^27^ found that BXM had no significant effects on clinical signs or histological scores. In contrast, in the present study, where the pathogenicity was less severe, we found that BXM initiated at 4 hpi was effective in reducing weight loss and histological scores at both low- and high-doses. The viral load in both nasal and tracheal swabs was reduced more effectively by BXM than oseltamivir in both the Suzuki study^27^ and ours, demonstrating its efficacy against the A(H7N9) virus. Taken together, these results indicate that BXM may provide a more effective treatment option than oseltamivir for A(H7N9) virus infection.

In the oseltamivir group, an NA-I26T- and NA-G133R-substituted virus was found in the nasal swab of one macaque on day 5. Oseltamivir-resistant mutations have been found in 23% of HPAI A(H7N9) viruses isolated from human cases treated with oseltamivir,^16^ but there have been no reports of resistance linked to NA substitutions 26 and/or 133 residues. In this study, we found that NA-I26T does not confer oseltamivir resistance and is located in the NA stalk region, far from the active site. NA-G133R (alone or in combination with NA-I26T) does not adequately support virus replication. This NA-I26T and NA-G133R-substituted virus was transient, and rapidly disappeared, suggesting that their impact may be limited. In the BXM group, PA-E319G and PA-K549R substitutions were observed at 1 dpi, but neither conferred BXM resistance and both disappeared by the next sampling (3 dpi). At 7 dpi, macaque #20 harboured viruses with different substitutions in different tissues: PA-E23G in the nasal swab and PA-I38T in the tracheal swab. Similarly, PA-K34R was detected in only one animal and at low titres. PA-I38T is a well-known BXM resistance substitution,^24^^,^^45^, ^46^, ^47^ listed by the WHO as a possible treatment-emergent substitution,^26^ and is the most frequent substitution known to reduce BXM susceptibility.^48^ PA-E23G and PA-K34R have also been reported as BXM resistance-associated substitutions,^49^, ^50^, ^51^ and are included in the WHO list.^26^ However, their EC_50_ values are low compared with that of I38T, as observed in this study, suggesting limited impact. These findings indicate that, under the conditions tested, BXM maintains potent antiviral activity against A(H7N9) viruses, although BXM-resistant viruses can emerge.

A limitation of this study is the relatively small sample size, which may limit its statistical power and contribute to inter-individual variability. In addition, infectious virus was detected in respiratory tissues only in a limited number of control animals at 7 dpi, likely reflecting sampling beyond the peak of viral replication. Despite these limitations, the overall findings from the longitudinal swab analyses and histopathological evaluation consistently demonstrated the antiviral efficacy of BXM. Based on the clear reduction in body weight loss, viral load, and tissue damage observed with early administration, BXM may contribute to controlling the spread and severity of HPAI A(H7N9) infections. BXM holds promise as a key component of the antiviral arsenal against highly pathogenic influenza viruses.

## Contributors

K.I-H., Masaki I., and Y.K. designed the experiments; K.I-H., Masaki I., T.I., Shinya Y., M.K., Mutsumi I., A.Y., and Seiya Y. performed the experiments; and K.I-H., T.I., and Y.K. wrote the manuscript. All authors read and approved the final version of the manuscript. K.I-H., Masaki I., T.I., Seiya Y., and Y.K. accessed and verified the underlying data.

## Data sharing statement

All data supporting the findings of this study are available in the paper. There are no restrictions to obtaining access to the primary data. Requests for data sharing should be directed to Yoshihiro Kawaoka (yoshihiro.kawaoka@wisc.edu).

Sequence data availability: The GISAID accession number for A/Guangdong/17SF003/2016-NA294R used in this study is EPI_ISL_20450171.

Code availability: No code was used in this study.

Reagent availability: BXM (S-033188) and oseltamivir phosphate were provided by Shionogi & Co., Ltd., and used for this study.

Other materials are available from the authors or from commercially available sources.

## Declaration of interests

T.I. was an employee of Shionogi Co., Ltd., and is a shareholder of Shionogi Co., Ltd. Y.K. has received unrelated grant support from Daiichi Sankyo Co., Ltd., Fujifilm Toyama Chemical Co., Ltd., Tauns Laboratories, Inc., Shionogi & Co. Ltd., Otsuka Pharmaceutical Co., Ltd., KM Biologics Co. Ltd., Kyoritsu Seiyaku Corporation, and Fuji Rebio, Inc. Y.K. is a co-founder of FluGen. The other authors do not have any competing interests.

## References

[1] Gao R., Cao B., Hu Y. (2013). Human infection with a novel avian-origin influenza A (H7N9) virus. N Engl J Med.

[2] Shen Y., Lu H. (2017). Global concern regarding the fifth epidemic of human infection with avian influenza A (H7N9) virus in China. Biosci Trends.

[3] Wang X., Jiang H., Wu P. (2017). Epidemiology of avian influenza A H7N9 virus in human beings across five epidemics in mainland China, 2013–17: an epidemiological study of laboratory-confirmed case series. Lancet Infect Dis.

[4] Zhou L., Ren R., Yang L. (2017). Sudden increase in human infection with avian influenza A (H7N9) virus in China, September–December 2016. West Pac Surveill Response J.

[5] Iuliano A.D., Jang Y., Jones J. (2017). Increase in human infections with avian influenza A(H7N9) virus during the fifth epidemic - china, October 2016-February 2017. MMWR (Morb Mortal Wkly Rep).

[6] Ke C., Mok C.K.P., Zhu W. (2017). Human infection with highly pathogenic avian influenza A(H7N9) virus, China. Emerg Infect Dis.

[7] Kile J.C. (2017). Update: increase in human infections with novel Asian lineage avian influenza A (H7N9) viruses during the fifth epidemic—China, October 1, 2016–August 7, 2017. MMWR (Morb Mortal Wkly Rep).

[8] Su S., Gu M., Liu D. (2017). Epidemiology, evolution, and pathogenesis of H7N9 influenza viruses in five epidemic waves since 2013 in China. Trends Microbiol.

[9] Zhang F., Bi Y., Wang J. (2017). Human infections with recently-emerging highly pathogenic H7N9 avian influenza virus in China. J Infect.

[10] Zhou L., Tan Y., Kang M. (2017). Preliminary epidemiology of human infections with highly pathogenic avian influenza A (H7N9) virus, China, 2017. Emerg Infect Dis.

[11] Yang Y., Wong G., Yang L. (2019). Comparison between human infections caused by highly and low pathogenic H7N9 avian influenza viruses in wave five: clinical and virological findings. J Infect.

[12] Yang L., Zhu W., Li X. (2017). Genesis and spread of newly emerged highly pathogenic H7N9 avian viruses in mainland China. J Virol.

[13] Shibata A., Okamatsu M., Sumiyoshi R. (2018). Repeated detection of H7N9 avian influenza viruses in raw poultry meat illegally brought to Japan by international flight passengers. Virology.

[14] Wu L., Mitake H., Kiso M. (2020). Characterization of H7N9 avian influenza viruses isolated from duck meat products. Transbound Emerg Dis.

[15] World Health Organization (2026). Avian influenza weekly update # 1037: 13 March 2026. https://www.who.int/westernpacific/publications/m/item/avian-influenza-weekly-update---1037--13-march-2026.

[16] Tang J., Zhang S.-X., Zhang J. (2020). Profile and generation of reduced neuraminidase inhibitor susceptibility in highly pathogenic avian influenza H7N9 virus from human cases in Mainland of China, 2016–2019. Virology.

[17] Imai M., Watanabe T., Kiso M. (2017). A highly pathogenic avian H7N9 influenza virus isolated from A human is lethal in some ferrets infected via respiratory droplets. Cell Host Microbe.

[18] O'Hanlon R., Shaw M.L. (2019). Baloxavir marboxil: the new influenza drug on the market. Curr Opin Virol.

[19] Hayden F.G., Sugaya N., Hirotsu N. (2018). Baloxavir marboxil for uncomplicated influenza in adults and adolescents. N Engl J Med.

[20] Ison M.G., Portsmouth S., Yoshida Y. (2020). Early treatment with baloxavir marboxil in high-risk adolescent and adult outpatients with uncomplicated influenza (CAPSTONE-2): a randomised, placebo-controlled, phase 3 trial. Lancet Infect Dis.

[21] Yen H.L., McKimm-Breschkin J.L., Choy K.T. (2013). Resistance to neuraminidase inhibitors conferred by an R292K mutation in a human influenza virus H7N9 isolate can be masked by a mixed R/K viral population. mBio.

[22] Sleeman K., Guo Z., Barnes J., Shaw M., Stevens J., Gubareva L. (2013). R292K substitution and drug susceptibility of influenza A(H7N9) viruses. Emerg Infect Dis.

[23] Marjuki H., Mishin V.P., Chesnokov A.P. (2015). Neuraminidase mutations conferring resistance to oseltamivir in influenza A(H7N9) viruses. J Virol.

[24] Omoto S., Speranzini V., Hashimoto T. (2018). Characterization of influenza virus variants induced by treatment with the endonuclease inhibitor baloxavir marboxil. Sci Rep.

[25] Takashita E., Kawakami C., Morita H. (2019). Detection of influenza A(H3N2) viruses exhibiting reduced susceptibility to the novel cap-dependent endonuclease inhibitor baloxavir in Japan, December 2018. Euro Surveill.

[26] World Health Organization (2024). Summary of polymerase acidic (PA) protein amino acid substitutions analysed for their effects on baloxavir susceptibility. https://www.who.int/publications/m/item/summary-of-polymerase-acidic-(pa)-protein-amino-acid-substitutions-analysed-for-their-effects-on-baloxavir-susceptibility.

[27] Suzuki S., Nguyen C.T., Ogata-Nakahara A. (2021). Efficacy of a cap-dependent endonuclease inhibitor and neuraminidase inhibitors against H7N9 highly pathogenic Avian influenza virus causing severe viral pneumonia in cynomolgus macaques. Antimicrob Agents Chemother.

[28] Shionogi & Co., Ltd (2018). Xofluza Tablets 10 mg, Xofluza Tablets 20 mg. https://www.pmda.go.jp/files/000225380.pdf.

[29] Watanabe T., Kawakami E., Shoemaker J.E. (2014). Influenza virus-host interactome screen as a platform for antiviral drug development. Cell Host Microbe.

[30] Neumann G., Watanabe T., Ito H. (1999). Generation of influenza A viruses entirely from cloned cDNAs. Proc Natl Acad Sci U S A.

[31] Heo Y.-A. (2018). Baloxavir: first global approval. Drugs.

[32] Yang T. (2019). Baloxavir marboxil: the first cap-dependent endonuclease inhibitor for the treatment of influenza. Ann Pharmacother.

[33] Schirmer P., Holodniy M. (2009). Oseltamivir for treatment and prophylaxis of influenza infection. Expert Opin Drug Saf.

[34] Kitano M., Itoh Y., Kodama M. (2011). Efficacy of single intravenous injection of peramivir against influenza B virus infection in ferrets and cynomolgus macaques. Antimicrob Agents Chemother.

[35] Itoh Y., Shinya K., Kiso M. (2009). In vitro and in vivo characterization of new swine-origin H1N1 influenza viruses. Nature.

[36] Watanabe T., Kiso M., Fukuyama S. (2013). Characterization of H7N9 influenza A viruses isolated from humans. Nature.

[37] Watanabe T., Shinya K., Watanabe S. (2011). Avian-type receptor-binding ability can increase influenza virus pathogenicity in macaques. J Virol.

[38] Kiso M., Iwatsuki-Horimoto K., Yamayoshi S. (2017). Emergence of oseltamivir-resistant H7N9 influenza viruses in immunosuppressed cynomolgus macaques. J Infect Dis.

[39] Koshimichi H., Retout S., Cosson V. (2020). Population pharmacokinetics and exposure-response relationships of baloxavir marboxil in influenza patients at high risk of complications. Antimicrob Agents Chemother.

[40] Ando Y., Noshi T., Sato K. (2021). Pharmacokinetic and pharmacodynamic analysis of baloxavir marboxil, a novel cap-dependent endonuclease inhibitor, in a murine model of influenza virus infection. J Antimicrob Chemother.

[41] Fukao K., Noshi T., Yamamoto A. (2019). Combination treatment with the cap-dependent endonuclease inhibitor baloxavir marboxil and a neuraminidase inhibitor in a mouse model of influenza A virus infection. J Antimicrob Chemother.

[42] Lee L.Y.Y., Zhou J., Frise R. (2020). Baloxavir treatment of ferrets infected with influenza A(H1N1)pdm09 virus reduces onward transmission. PLoS Pathog.

[43] Aoki F.Y., Macleod M.D., Paggiaro P. (2003). Early administration of oral oseltamivir increases the benefits of influenza treatment. J Antimicrob Chemother.

[44] Heinonen S., Silvennoinen H., Lehtinen P. (2010). Early oseltamivir treatment of influenza in children 1-3 years of age: a randomized controlled trial. Clin Infect Dis.

[45] Chesnokov A., Patel M.C., Mishin V.P. (2020). Replicative fitness of seasonal influenza A viruses with decreased susceptibility to baloxavir. J Infect Dis.

[46] Checkmahomed L., M'Hamdi Z., Carbonneau J. (2020). Impact of the baloxavir-resistant polymerase acid I38T substitution on the fitness of contemporary influenza A(H1N1)pdm09 and A(H3N2) strains. J Infect Dis.

[47] Imai M., Yamashita M., Sakai-Tagawa Y. (2020). Influenza A variants with reduced susceptibility to baloxavir isolated from Japanese patients are fit and transmit through respiratory droplets. Nat Microbiol.

[48] Takashita E. (2021). Influenza polymerase inhibitors: mechanisms of action and resistance. Cold Spring Harb Perspect Med.

[49] Gubareva L.V., Mishin V.P., Patel M.C. (2019). Assessing baloxavir susceptibility of influenza viruses circulating in the United States during the 2016/17 and 2017/18 seasons. Euro Surveill.

[50] Takashita E., Daniels R.S., Fujisaki S. (2020). Global update on the susceptibilities of human influenza viruses to neuraminidase inhibitors and the cap-dependent endonuclease inhibitor baloxavir, 2017-2018. Antivir Res.

[51] Govorkova E.A., Takashita E., Daniels R.S. (2022). Global update on the susceptibilities of human influenza viruses to neuraminidase inhibitors and the cap-dependent endonuclease inhibitor baloxavir, 2018–2020. Antivir Res.

