# Supplementary material for: Effectiveness of baloxavir marboxil in nonhuman primates infected with highly pathogenic avian influenza A(H7N9) virus
Source: eBioMedicine. 2026 Jun 25;129:106350. doi: 10.1016/j.ebiom.2026.106350 (PMC13325332; doi:10.1016/j.ebiom.2026.106350)
Supplement: Supplementary Tables and Figure [file mmc1.pdf]

Supplementary Table 1. Reagent Validation

Reagent	Supplier	Catalog No.	RRID	Application	Validation
Pan Influenza A Nucleoprotein Antibody, Rabbit MAb	Sino Biological	40208-R010	AB_3677053	IHC	Although the manufacturer's data sheet lists western blotting and ELISA applications, the suitability of this antibody for IHC in formalin-fixed paraffin-embedded tissues was confirmed prior to use in this study by Sept.Sapie Co., Ltd.
Anti-IgG(γ), Monkey, Goat-poly, HRP	Funakoshi	5220-0333	-	ELISA	Validated by the manufacturer for ELISA
Human Anti-Influenza virus A IgG ELISA Kit	abcam plc	ab108745	-	ELISA	Validated by the manufacturer for ELISA

Cell line	Source	Mycoplasma status	Authentication
MDCK	Laboratory stock	Negative	Confirmed by DNA fingerprinting analysis against ATCC controls

Supplementary Table 2. Grouping of animals

Animal #	Treatment	Dose (mg/kg)					Treatment started at	Observation period
		1st	2nd	3rd	4th	5th		
#1-3	vehicle	-	-	-	-	-	4 hours	21 days
#4-6	low-dose BXM	4.5	1.5	1.2	0.8	0.6		
#7-9	high-dose BXM	7.0	2.8	2.1	8.6	4.0		
#10-12	oseltamivir	100	100	100	100	100		
#13-15	vehicle	-	-	-	-	-	4 hours	7 days
#16-18	low-dose BXM	4.5	1.5	1.2	0.8	0.6		
#19-21	high-dose BXM	7.0	2.8	2.1	8.6	4.0		
#22-24	oseltamivir	100	100	100	100	100		
#25-27	vehicle	-	-	-	-	-	48 hours	21 days
#28-30	low-dose BXM	4.5	1.5	1.2	0.8	0.6		
#31-33	high-dose BXM	7.0	2.8	2.1	8.6	4.0		
#34-36	oseltamivir	100	100	100	100	100		
#37-39	vehicle	-	-	-	-	-	48 hours	7 days
#40-42	low-dose BXM	4.5	1.5	1.2	0.8	0.6		
#43-45	High-dose BXM	7.0	2.8	2.1	8.6	4.0		
#46-48	oseltamivir	100	100	100	100	100		

Supplementary Table 3. Clinical signs monitored in cynomolgus macaques following infection.

Observation type	Parameter	Clinical sign monitored
Visual observation from outside the cage	Nutritional status	decreased appetite or anorexia
	Posture	Sitting, lateral recumbency, prone position, crouching
	Level of consciousness	Sedation, somnolence, coma
	Respiratory signs	Tachypnea, bradypnea, deep breathing, shallow breathing, dyspnea
	Eyes	Ocular discharge (lacrimation, reddish tears, ocular discharge); amount and characteristics
	Ears	Response to sound, presence of discharge, erythema of the auricle
	Nose	Presence and characteristics of nasal discharge, dryness, abnormal sounds, bleeding
	Body surface	Coat condition (gloss, contamination, piloerection, alopecia), erosion, erythema or pallor of the skin
	Gait abnormalities	Ataxic gait, paralytic gait, difficulty walking
	Reflexes	Righting reflex, auricular reflex, corneal reflex, pupillary light reflex
	Tremor	Location of tremor
	Convulsions	Clonic or tonic convulsions
	Urination	Frequency, incontinence, color, turbidity, volume, odor
	Feces	Color and consistency (soft stool, diarrhea, mucus stool, tarry stool, bloody stool, abnormal color)
	Vomiting	Frequency, characteristics (food-like material, mucous, watery), volume, color
	Bleeding	Menstrual bleeding
Observation during handling (visual and tactile examination)	Eyes	Ocular discharge; corneal condition (opacity, neovascularization); eyelid condition (including nictitating membrane; partial closure, ptosis); exophthalmos or enophthalmos; conjunctival or scleral color changes (erythema, pallor); iris abnormalities (reduced pupillary reflex, miosis, mydriasis)
	Ears	Presence of discharge
	Nose	Presence and characteristics of nasal discharge, dryness, bleeding
	Oral cavity	Erythema or pallor of the oral mucosa, ulcers
	Body surface	Coat condition (gloss, contamination, piloerection, alopecia), erosion, erythema or pallor, swelling of lymph nodes or skin, masses, muscle tone
	Body temperature	Hypothermia or hyperthermia
	External genitalia	Urine contamination of the lower abdomen, contamination around the anus
	Salivation	Amount (mild, moderate, severe)
	Other	Skin color, presence of wounds, lymph node or skin swelling or masses, muscle tone, heart rate, respiratory status

Supplementary Table 4. Clinical signs in H7N9 virus-infected cynomolgus macaques that began treatment 4 hours post-infection.

	Low-dose BXM						High-dose BXM						Oseltamivir						Vehicle					
Day	#4	#5	#6	#16	#17	#18	#7	#8	#9	#19	#20	#21	#10	#11	#12	#22	#23	#24	#1	#2	#3	#13	#14	#15
0	0	0	0	0	0	0	0	0	0	0	0	0	0	0	0	0	0	0	0	0	0	0	0	0
1	0	0	0	0	0	0	0	0	0	0	0	0	0	0	0	0	0	0	0	0	0	0	0	0
2	0	0	0	0	0	0	0	0	0	0	0	0	0	0	0	0	0	0	0	0	0	0	0	0
3	0	0	0	0	0	0	0	0	0	0	0	0	0	0	0	0	0	0	0	0	0	0	0	0
4	0	0	0	0	0	0	0	0	0	0	0	0	0	0	0	0	0	0	0	0	0	0	0	0
5	0	0	0	0	0	0	0	0	0	0	0	0	0	0	0	0	0	0	0	0	0	0	0	0
6	0	0	0	0	0	0	0	0	0	0	0	0	0	0	0	0	0	0	0	0	0	0	0	0
7	0	0	0	0	0	0	0	0	0	0	0	0	0	0	0	0	0	0	0	0	0	0	0	0
8	0	0	0	—[a]	—	—	0	0	0	—	—	—	0	0	0	—	—	—	0	0	0	—	—	—
9	0	0	0	—	—	—	0	0	0	—	—	—	0	0	0	—	—	—	0	0	0	—	—	—
10	0	0	0	—	—	—	0	0	0	—	—	—	0	0	0	—	—	—	0	0	0	—	—	—
11	0	0	0	—	—	—	0	0	0	—	—	—	0	0	0	—	—	—	0	0	0	—	—	—
12	0	0	0	—	—	—	0	0	0	—	—	—	0	0	0	—	—	—	0	0	0	—	—	—
13	0	0	0	—	—	—	0	0	0	—	—	—	0	0	0	—	—	—	0	0	0	—	—	—
14	0	0	0	—	—	—	0	0	0	—	—	—	0	0	0	—	—	—	0	0	0	—	—	—
15	0	0	0	—	—	—	0	0	0	—	—	—	0	0	0	—	—	—	0	0	0	—	—	—
16	0	0	0	—	—	—	0	0	0	—	—	—	0	0	0	—	—	—	0	0	0	—	—	—
17	0	0	0	—	—	—	0	0	0	—	—	—	0	0	0	—	—	—	0	0	0	—	—	—
18	0	0	0	—	—	—	0	0	0	—	—	—	0	0	0	—	—	—	0	0	0	—	—	—
19	0	0	0	—	—	—	0	0	0	—	—	—	0	0	0	—	—	—	0	0	0	—	—	—
20	0	0	0	—	—	—	0	0	0	—	—	—	0	0	0	—	—	—	0	0	0	—	—	—
21	0	0	0	—	—	—	0	0	0	—	—	—	0	0	0	—	—	—	0	0	0	—	—	—

Clinical signs were observed twice daily until Day 9 and once daily thereafter. 0: no abnormal signs

[a] —, animal not available

Supplementary Table 5. Clinical signs in H7N9 virus-infected cynomolgus macaques that began treatment 48 hours post-infection.

	Low-dose BXM						High-dose BXM						Oseltamivir						Vehicle					
Day	#28	#29	#30	#40	#41	#42	#31	#32	#33	#43	#44	#45	#34	#35	#36	#46	#47	#48	#25	#26	#27	#37	#38	#39
0	0[a]	0	0	0	0	0	0	0	0	0	0	0	0	0	0	0	0	0	0	0	0	0	0	0
1	0	0	0	0	0	0	0	0	0	0	0	0	0	0	0	0	0	0	0	0	0	0	0	0
2	0	0	0	0	0	0	0	0	0	0	0	0	0	0	0	0	0	0	0	0	0	0	0	0
3	0	0	0	0	0	0	0	0	0	0	0	0	0	0	0	0	0	0	0	0	0	0	0	0
4	0	0	0	0	0	0	0	0	0	0	0	0	0	0	0	0	0	0	0	0	0	0	0	0
5	0	0	0	0	0	0	0	0	0	0	0	0	0	0	0	0	0	0	0	0	0	1:aB[b]<br>+:oz[c]	0	0
6	0	0	0	0	0	0	0	0	0	0	0	0	0	0	0	0	0	0	0	0	0	0	0	0
7	0	0	0	0	0	0	0	0	0	0	0	0	0	0	0	0	0	0	0	0	0	0	0	0
8	0	0	0	—[a]	—	—	0	0	0	—	—	—	0	0	0	—	—	—	0	0	0	—	—	—
9	0	0	0	—	—	—	0	0	0	—	—	—	0	0	0	—	—	—	0	0	0	—	—	—
10	0	0	0	—	—	—	0	0	0	—	—	—	0	0	0	—	—	—	0	0	0	—	—	—
11	0	0	0	—	—	—	0	0	0	—	—	—	0	0	0	—	—	—	0	0	0	—	—	—
12	0	0	0	—	—	—	0	0	0	—	—	—	0	0	0	—	—	—	0	0	0	—	—	—
13	0	0	0	—	—	—	0	0	0	—	—	—	0	0	0	—	—	—	0	0	0	—	—	—
14	0	0	0	—	—	—	0	0	0	—	—	—	0	0	0	—	—	—	0	0	0	—	—	—
15	0	0	0	—	—	—	0	0	0	—	—	—	0	0	0	—	—	—	0	0	0	—	—	—
16	0	0	0	—	—	—	0	0	0	—	—	—	0	0	0	—	—	—	0	0	0	—	—	—
17	0	0	0	—	—	—	0	0	0	—	—	—	0	0	0	—	—	—	0	0	0	—	—	—
18	0	0	0	—	—	—	0	0	0	—	—	—	0	0	0	—	—	—	0	0	0	—	—	—
19	0	0	0	—	—	—	0	0	0	—	—	—	0	0	0	—	—	—	0	0	0	—	—	—
20	0	0	0	—	—	—	0	0	0	—	—	—	0	0	0	—	—	—	0	0	0	—	—	—
21	0	0	0	—	—	—	0	0	0	—	—	—	0	0	0	—	—	—	0	0	0	—	—	—

Clinical signs were observed twice daily until Day 9 and once daily thereafter. 0: no abnormal signs, 1: slight, 2: moderate, 3: severe, +: non-graded

[a] —, animal not available

[b] aB, decrease in spontaneous activity

[c] oz, delayed awakening from anesthesia

These signs were observed only during the afternoon monitoring session.

Supplementary Table 6. Immunohistochemistry with influenza A nucleoprotein antibody in the lungs of H7N9 virus-infected cynomolgus macaques.

	Treatment started at 4 hpi												Treatment started at 48 hpi											
	Low-dose BXM			High-dose BXM			Oseltamivir			Vehicle			Low-dose BXM			High-dose BXM			Oseltamivir			Vehicle		
Cell type	#16	#17	#18	#19	#20	#21	#22	#23	#24	#13	#14	#15	#40	#41	#42	#43	#44	#45	#46	#47	#48	#37	#38	#39
Bronchial epithelial cells	0	0	0	0	1	0	1	0	0	1	1	0	1	1	0	1	1	1	1	1	1	1	1	1
Endobronchial macrophages	0	0	0	0	0	0	0	0	0	0	0	0	0	0	0	0	0	0	0	0	0	0	0	0
Bronchiolar epithelial cells	0	0	0	0	1	0	0	0	0	0	0	1	1	1	1	0	1	0	1	0	0	1	1	0
Alveolar macrophages	1	1	1	0	1	1	2	0	0	1	1	2	1	2	1	1	1	1	2	0	0	2	1	0
Alveolar epithelial cells	1	0	2	2	2	2	2	1	0	0	0	1	2	2	1	2	1	1	2	1	1	2	2	1

Cells were scored semi-quantitatively for immunopositivity. 0: negative, 1: very slight, 2: slight, 3: moderate, 4: marked

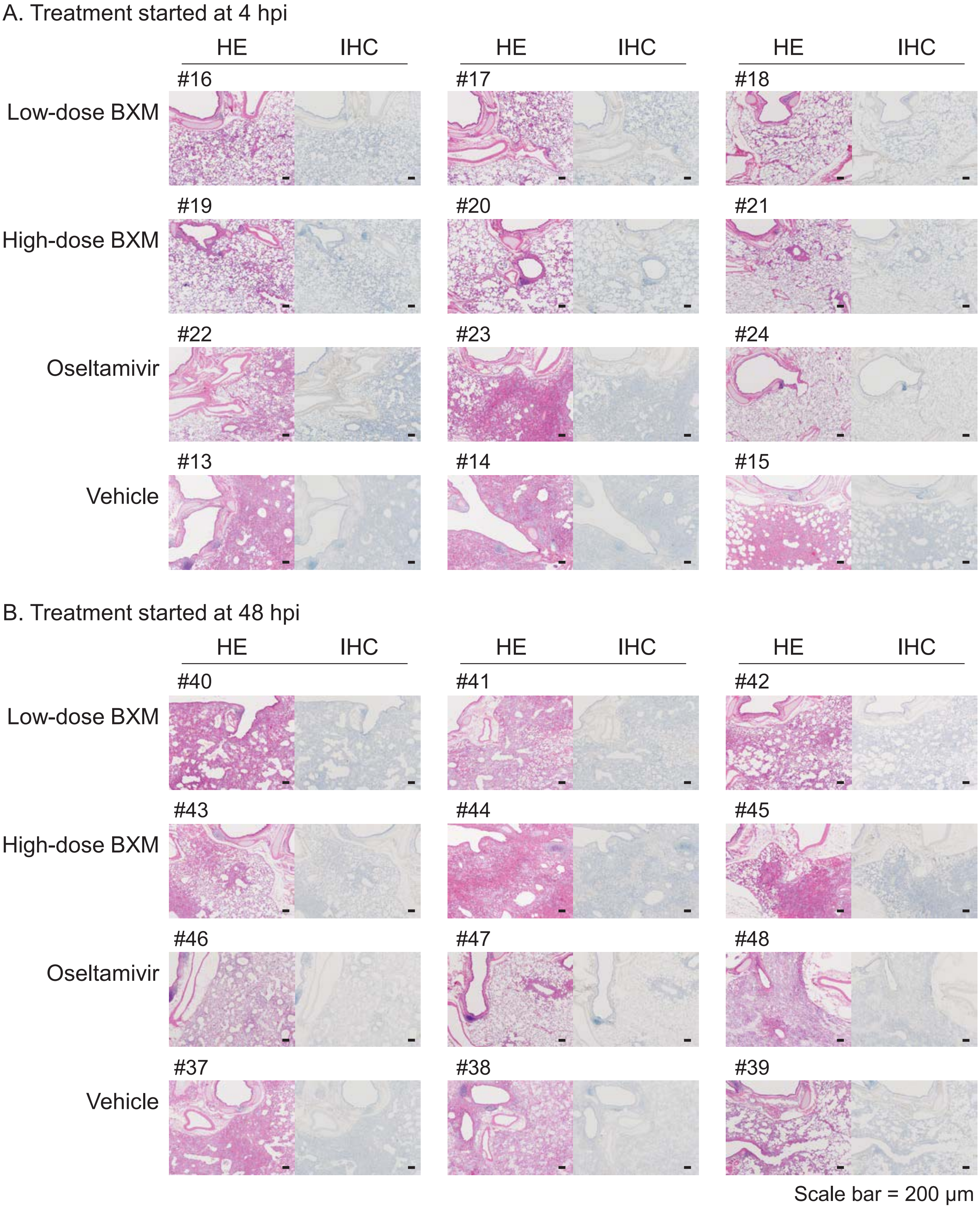

Supplementary Fig. 1. Pathological examination of the lungs of macaques.
The images show the hematoxylin and eosin staining (HE) and immunohistochemical (IHC) analysis of the lungs of macaques at 7 dpi in the low- or high-dose BXM, oseltamivir , or control (vehicle) groups, with treatment started at 4 hpi (A) or 48 hpi (B). Scale bars = 200 μm.
